# Clinical Notes on Cancer

**Published:** 1883-12

**Authors:** 


*Clinical Notes on Cancer.* By HERBERT L. SNOW, M.D. Lond., and Surgeon to the Cancer Hospital, Brompton. Pp. 96. London: J. & A. Churchill. 1883.

In this work, as the author himself says in the preface, "no attempt has been made at elaborate description or exhaustive investigation," and the result is just such as might have been expected. If the endeavours to avoid the elaborate only lead to a falling into the inaccurate and the involved (pp. 14 and 15), and an eschewing of exhaustiveness in investigation results in nothing more than a record of vague "impressions," then we think the author had better not have written a book. It would be easy, if space permitted, to quote whole paragraphs as specimens of logical fallacies. He ventures (as he says) upon a "feeble attempt" *(sic)* to controvert the Heredity Fallacy, and the result does not transcend the author's expectations. Speaking of breast-scirrhus, he refers to a peculiar condition where there "is a tenderness on pressure, often combined with perceptible enlargement, in the head and neck of the humerus which corresponds to the disease. . . . This may disappear (both enlargement and tenderness) after operation." Now, no statement such as this ought to be made by a scientific man without irrefragable pathological proof in its support. If it is inflammatory œdema, then it is not enlargement of the head and neck of the humerus; and if it is bony enlargement, then it is cancerous and will not disappear after operation. But hear what he says in the next sentence:—"It would seem to be the first manifestations of that implication of the osseous system which becomes so painfully evident in many cases of advanced scirrhus," and excision of the breast may cure it!

The practical part of the work is on a level with the theoretical, and certainly not abreast of the highest class practice of to-day. In uterine cancer, for instance, if potassa fusa and perchloride of iron fail, and he is granted a "long cervix and disease limited to the margin of the os uteri," he would use the galvanic cautery. This is the limit of his resources. Has he never heard of supravaginal amputation of the cervix, or of extirpation of the whole uterus?

The work is one more example of how (to use the author's own words) "impressions received through constant familiarity with common every-day cases," such as specialists see in special hospitals, may be absolutely impotent for the furtherance of science.

